# Vision-related quality of life and symptom perception change over time in newly-diagnosed primary open angle glaucoma patients

**DOI:** 10.1038/s41598-019-43203-9

**Published:** 2019-05-01

**Authors:** Ivano Riva, Lorenzo Legramandi, Eliana Rulli, Anastasios G. Konstas, Andreas Katsanos, Francesco Oddone, Robert N. Weinreb, Luciano Quaranta, The Italian Study Group on QoL in Glaucoma, L. Varano, T. Carchedi, S. Talarico, F. Parravano, I. Motolese, S. A. Bagaglia, G. C. M. Rossi, S. Lateri, L. Bossolesi, L. Carmassi, T. Rolle, R. Piccini, R. Ratiglia, A. Rossi, S. Gandolfi, Tagliavini, N. Ungaro, M. Fossarello, A. Cuccu, I. Zucca, M. Uva, E. Bonacci, G. Cardarella, D. Tognetto, O. Vattovani, P. Vallon, F. Iannacone, L. Fontana, S. Marchi, G. L. Manni, D. Jannetta, G. Roberti, L. Rossetti, E. Maggiolo, O. Oneta, C. Sborgia, F. Cantatore, L. Mastropasqua, L. Agnifili, E. Campos, C. Gizzi, G. Giannaccare, Pucci, M. Cassamali, C. Costagliola, C. Traverso, R. Scotto, M. Musolino, L. Landi, A. Bagnis

**Affiliations:** 1 IRCCS - Fondazione Bietti, Rome, Italy; 2 IRCCS - Istituto di Ricerche Farmacologiche Mario Negri, Milan, Italy; 3 1st University Department of Ophthalmology, Aristotle University of Thessaloniki, Thessaloniki, Greece; 4 Department of Ophthalmology, University of Ioannina, Ioannina, Greece; 5 Hamilton Glaucoma Center, Shiley Eye Institute, and the Department of Ophthalmology, University of California San Diego, San Diego, USA; 6 Department of Surgical & Clinical, Diagnostic and Pediatric Sciences, Section of Ophthalmology, University of Pavia - IRCCS Fondazione Policlinico, San Matteo, Pavia Italy; 7 Università degli Studi “Magna Graecia”, Catanzaro, Italy; 8 A.O.U. Senese Ospedale Santa Maria delle Scotte, Università di Siena, Siena, Italy; 9 Fondazione IRCCS Istituto di Ricovero e Cura a Carattere Scientifico Policlinico San Matteo, Pavia, Italy; 10 IRCCS Istituto di Ricovero e Cura a Carattere Scientifico Istituto Auxologico Italiano, Milano, Italy; 11 Università degli Studi di Torino, Torino, Italy; 12 Fondazione IRCCS Istituto di Ricovero e Cura a Carattere Scientifico Ca’ Granda Ospedale Maggiore Policlinico, Milano, Italy; 13 A.O.U. di Parma, Parma, Italy; 14 A.O.U. Cagliari - Ospedale Civile San Giovanni di Dio, Cagliari, Italy; 15 A.O.U. “Policlinico Vittorio Emanuele” P.O. Gaspare Rodolico, Catania, Italy; 16 A.O.U. “Ospedale Maggiore”, Trieste, Italy; 17 A.O. Arcispedale Santa Maria Nuova-IRCCS, Reggio Emilia, Italy; 18 Università Tor Vergata, Fondazione Policlinico Tor Vergata, Roma, Italy; 19 A.O. S. Paolo, Milano, Italy; 20 A.O.U. Policlinico, Bari, Italy; 21 Ospedale Clinicizzato SS. Annunziata, Chieti, Italy; 22 A.O.U. Policlinico S. Orsola Malpighi, Bologna, Italy; 23 A.O. di Desenzano del Garda, Desenzano del Garda, Brescia, Italy; 24 Dipartimento SPES Università del Molise, Campobasso, Italy; 25 IRCCS AOU San Martino - IST, Genova, Italy

**Keywords:** Medical research, Diseases

## Abstract

To evaluate the change over time of vision-related quality of life (QoL) and glaucoma symptoms in a population of newly-diagnosed primary open angle glaucoma (POAG) patients. Multicenter, prospective study. Consecutive newly-diagnosed POAG patients were enrolled and followed-up for one year. Follow-up visits were scheduled at 6 and 12 months from baseline. At each visit, vision-related QoL and glaucoma-related symptoms were assessed by the means of the 25-item National Eye Institute Visual Function Questionnaire (NEI-VFQ-25) and the Glaucoma Symptom Scale (GSS), respectively. Trends over time for NEI-VFQ-25 and GSS scores were evaluated with longitudinal linear mixed models. One-hundred seventy-eight patients were included in the analysis. At baseline, early to moderate glaucoma stages were associated with higher scores for most GSS and NEI-VFQ-25 items, while lower best-corrected visual acuity was associated with lower scores for 4 of the 12 NEI-VFQ-25 items. During the follow-up, all the GSS scores, the NEI-VFQ-25 total score, and 7 of the 12 NEI-VFQ-25 scores significantly improved (p < 0.05). In multivariate model, higher increases of most GSS and NEI-VFQ-25 scores were modeled in patients with low scores at baseline. Vision-related QoL and glaucoma-related symptom perception significantly improved during the one-year follow-up in this population of newly diagnosed POAG patients.

## Introduction

Glaucoma, one of the leading causes of blindness in the world^1^, is characterized by progressive visual field (VF) deterioration and optic disc damage^2^. Primary open angle glaucoma (POAG), the most common type of glaucoma in white populations^3^, has a chronic course with functional impairment developing over years. Although glaucoma is an irreversible disease, its progression may be delayed with appropriate medical and surgical management^4–6^.

In recent years, quality of life (QoL) has assumed a leading role in health-care, and its preservation is now one of the main goals of treatment^7,8^. As a chronic disease, POAG may affect QoL^9–14^. VF loss and central visual acuity impairment may limit daily activities, such as reading^15,16^, walking^17^ or driving^18,19^. Additionally, topical medications^20,21^, surgical interventions^22,23^ and the need for long-term care are additional factors that can significantly affect QoL.

Several studies have evaluated QoL in glaucoma and the association with its clinical aspects, such as VF damage (VFD)^9,10,24,25^ and best corrected visual acuity (BCVA) deterioration^24,26^, or the presence of unilateral versus bilateral disease^27,28^. A significant correlation between disease severity and the impairment of QoL has been found using either generic or vision-specific instruments^14^. However, the majority of these studies had a cross-sectional design, whereby data collected at a single time-point were analyzed. Differently, the Collaborative Initial Glaucoma Treatment Study (CIGTS) prospectively evaluated QoL changes in newly-diagnosed glaucoma patients, randomized to medical or surgical treatment, over a 5-year follow-up^23^. As a result, the overall trend was a decline in the percent of participants of both groups reporting the presence of functional and local symptoms over time, suggesting an improvement of patient’s QoL perception. However, the CIGTS used a proprietary multidimensional instrument developed by the CIGTS investigators, and interviews were performed by phone. No other study subsequently applied the same QoL instrument.

The Italian Primary Open Angle Glaucoma Study (IPOAGS) is a multicenter study designed to evaluate vision-related QoL in Italian POAG patients^29–31^. The IPOAGS consists of a cross-sectional and a prospective part. The present report describes the results of the prospective part, the aim of which was to assess possible changes in vision-related QoL and symptom perception over time, and to identify patterns of QoL modification according to the characteristics of the disease.

## Material and Methods

The design and methods of the IPOAGS have been described elsewhere^29–31^. Briefly, previously or newly diagnosed POAG patients aged 18 or more were consecutively enrolled during routine visits at 21 academic and non-academic centers in Italy. In the cross-sectional part of the study, vision-related QoL data were collected and their association with both clinical and socio-demographic variables was investigated^29,30^. In the prospective part of the study, consecutive newly-diagnosed glaucoma patients were evaluated at baseline and were followed-up for one year.

Informed consent was obtained from all the participants before any study-related procedure and after the nature and the purpose of the investigation were fully explained. The study was conducted in accordance with the tenets of the Declaration of Helsinki and the Guidelines for Good Clinical Practice. The Institutional Review Board of Brescia (Brescia, Italy) and of each participating center approved the protocol. The study was registered at clinicaltrials.gov (NCT01742104).

### Interventions

Newly-diagnosed POAG patients underwent a comprehensive ophthalmological examination at baseline and at each follow-up visit. Follow-up study visits were scheduled at 6 and 12 months from baseline, however interim visits were allowed at clinician discretion, to adjust therapy and evaluate target IOP. Treatment was initiated, at the end of the baseline visit, to avoid an influence of drugs and interventions on QoL assessment. At baseline and at 6- and 12-month follow-up visits, VF examination was performed and vision-related QoL and glaucoma symptom perception questionnaires were administered.

POAG was defined as a typical optic neuropathy with a focal, generalized or focal/generalized neuroretinal thinning or an inter-eye cup/disc ratio asymmetry >0.2. The presence of typical glaucomatous VF defects was not a prerequisite for inclusion, if the investigator’s clinical judgment was strongly in favor of POAG (e.g. due to high intraocular pressure (IOP), retinal nerve fiber layer defects or optic disc hemorrhages)^32^.

Vision-related QoL was assessed using the validated Italian version of the 25-item National Eye Institute Visual Function Questionnaire (NEI-VFQ-25)^33^, while glaucoma-related symptoms were assessed using the validated Italian version of the Glaucoma Symptom Scale questionnaire (GSS)^34^.

The NEI-VFQ-25 is a self-administered questionnaire, developed to investigate vision-related QoL^35^. It consists of 25 vision-targeted questions, representing 11 vision-related constructs (general vision, ocular pain, near activities, distance activities, social functioning, mental health, role difficulties, dependency, driving, color vision and peripheral vision), plus an additional single-item question for general health rating. The scoring procedure converts the pre-coded numeric values of each item to a score ranging from 0 to 100. Higher scores reflect better vision-related QoL. The total score is the mean of all the vision-targeted subscales scores.

The GSS is a glaucoma-specific tool designed for the investigation of symptoms in patients with glaucoma^36^. It was developed as a modified instrument from a checklist of symptoms applied in the Ocular Hypertension Treatment Study^36^. It includes 10 items grouped in two domains: Symp-6 for non-visual symptoms (burning/smarting/stinging, tearing, dryness, itching, soreness/tiredness, feeling of something in the eye) and Func-4 for visual symptoms (blurry/dim vision, hard to see in daylight, hard to see in darkness, and halos around the lights). Each item can take a score from 0 to 100, where 0 indicates very bothersome symptoms and 100 the absence of symptoms. The domain score is the mean from the items of the relative domain, while the total GSS score is the mean from all items of both domains. Each eye is tested separately.

### Visual field and stage of the disease

A reliable VF examination at baseline (i.e. false-positive responses <15% with a clear blind-spot at the VF printout, threshold value <10 dB) was required for study inclusion. Severity of VF impairment was classified at baseline and at each follow-up visit according to the Glaucoma Staging System 2 (GStag2)^37,38^. GStag2 is a classification method using Mean Deviation (MD) or Mean Defect and Pattern Standard Deviation/Corrected Pattern Standard Deviation (PSD/CPSD) and Loss Variation/Corrected Loss Variation (LV/CLV) values (from either the 30-2/24-2 Zeiss-Humphrey tests or the G1/G1X/G2 Octopus programs) to create a Cartesian coordinate diagram. VF is classified into seven different stages by curvilinear lines from stage 0 (normal VF) to stage 5 (low threshold readings, with only small remnants of sensitivity). GStag2 has been validated in population-based studies^39–43^ and has been shown to be similar or superior to other VF classification systems^44^. The improvement of performance in perimetric tests is a well-known phenomenon, called “learning effect”. To prevent bias, the “worst value carry backward” method was used: for each eye, the highest value of glaucoma stage during the follow-up replaced the earlier values.

### Statistical analysis

All the tests were performed considering patient as unit of analysis. In patients with bilateral disease, arithmetical means of BCVA and MD were computed, while the lowest GSS score and the worst stage of the disease were chosen.

For statistical purposes, stage of the disease was categorized as follows: early (stage 0, borderline and 1), moderate (stage 2 and 3) and severe (stage 4 and 5). BCVA was dichotomized as ≤ or >0.15 LogMar. Since the information on prescribed treatments were collected at fixed time-points (baseline, 6 and 12-month visits), but treatment variation was at clinician’s discretion during the follow-up, data from 6-month visit were taken as a representative proxy of the entire follow-up.

Vision-related QoL and symptom scores expressed as a mean of different subscale scores (e.g. total NEI-VFQ-25 or total GSS scores) were treated as continuous variables. Single-item scores and scores with a value of 100 for more than 50% of the interviewed subjects were treated as dichotomous variables. In this case, “1” was assigned to the maximum score of 100, and “0” to other values. Dichotomized variables, exclusively for the NEI-VFQ-25 were: distance activities, color vision, peripheral vision, social functioning, role difficulties and dependency.

NEI-VFQ-25 and GSS scores at baseline were investigated by the means of multivariable general linear models and logistic models. Vision-related QoL and symptom scores over time were modeled as a linear function of time. A multivariable analysis including time, baseline QoL scores and their interaction with time and covariates was performed. The adjustment for baseline QoL scores and interaction with time was performed taking into account regression toward the mean and ceiling effect. Longitudinal linear mixed models and generalized longitudinal mixed models for binary data with a marginal logit link function (modeled the probability of score = “100”) were used for the analyses of dichotomized scores. The effect of time was modeled as a linear function. Random intercepts and random slopes for time effect were introduced to interpolate the subject-specific deviations from the score trends.

A significance level of p < 0.05 was set. All the analyses were performed with SAS software (version 9.4).

## Results

From March 2012 to July 2013, one hundred ninety-seven newly-diagnosed POAG patients were enrolled. Nineteen patients were then excluded from the analysis, 11 because they did not have follow-up data and 8 because they did not have data on anti-glaucoma prescriptions. Therefore, analyses were performed on 178 subjects.

Demographic characteristics of the study participants are presented in Table 1. Mean age was 61.7 years (SD 14.3), and 52.8% of the participants were females. Mean values for MD and BCVA were −4.5 dB (SD 5.3) and 0.1 LogMar (SD 0.1). According to the GStag2, disease severity was classified as early (stage 0, borderline ad 1) in 95 patients (53.3%), moderate (stage 2 and 3) in 56 patients (31.5%) and severe (stage 4 and 5) in 27 patients (15.2%). After the baseline visit, all the patients were administered one or more hypotensive topical medications (Table 2). Eighty-nine patients (50%) were administered a prostaglandin analogue alone, while a β-blocker was administered in 53 patients (29.8%). Thirty patients (16.8%) received more than one topical agent as a first-line therapy. During the follow-up, 8 patients (4.4%) underwent surgery and 27 patients received laser treatment (i.e. selective laser trabeculoplasty, 15.2%), in addition to medical therapy.

**Table 1:** Demographic and clinical characteristics of study participants at baseline.

	Overall N = 178
Age–Mean (SD)	61.7 (14.3)
Male sex–n (%)	84 (47.2)
Visual field MD (dB) - Mean (SD)	−4.5 (5.3)
BCVA (LogMar)–Mean (SD)	0.1 (0.1)
Stage of the disease*–n (%)	
Stage 0	2 (1.1)
Border	47 (26.4)
Stage 1	46 (25.8)
Stage 2	35 (19.7)
Stage 3	21 (11.8)
Stage 4	10 (5.6)
Stage 5	17 (9.6)

Legend: N: total number of participants; SD: Standard Deviation; MD: Mean Deviation; dB: Decibel

BCVA: Best Corrected Visual Acuity. *According to Glaucoma Staging System 2.

**Table 2:** Treatments of patients included in the study.

	Overall N = 178			
Intervention – n (%)				
Topical, surgical and laser	4 (2.2)			
Topical and Surgical	4 (2.2)			
Topical and Laser	27 (15.2)			
Only Topical	143 (80.3)			
**Topical Treatments – n (%)**	**Baseline**	**6 months**	**12 months**	
β-blockers	54 (30.3)	46 (25.8)	47 (26.4)	
Carbonic anhydrase inhibitors	7 (3.9)	7 (3.9)	12 (6.7)	
Prostaglandin analogues	96 (53.9)	108 (60.7)	100 (56.2)	
Parasympathomimetics	0 (0.0)	1 (0.6)	1 (0.6)	
Prostaglandin + beta-blocker FC	15 (8.4)	16 (9.0)	26 (14.6)	
Alpha agonist + beta-blocker FC	1 (0.6)	1 (0.6)	1 (0.6)	
Carbonic anhydrase inhibitor + β-blocker FC	12 (6.7)	18 (10.1)	15 (8.4)	
Other	2 (1.1)	2 (1.1)	2 (1.1)	
**Number of active agents – n (%)**	**Baseline**	**6 months**	**12 months**	**P-value** ^**a**^
0	0 (0.0)	1 (0.6)	5 (2.8)	<0.01
1	148 (83.1)	133 (74.7)	120 (67.9)	
2	23 (12.9)	31 (17.4)	35 (19.7)	
≥3	7 (3.9)	13 (7.3)	18 (10.1)	

Legend: N: Total number of participants; FC: Fixed combination; ^a^Friedman test.

### Vision-related QoL scores at baseline

Results of multivariable analyses on baseline QoL scores are showed in Table 3. Being a man (p = 0.03) and having an early/moderate vs. severe stage of the disease (p < 0.01) were variables associated with higher values of GSS Func-4 score at baseline. Early or moderate vs. severe stages of the disease were associated with higher values of most NEI-VFQ-25 scores, with the exception of general health (p = 0.06) and ocular pain (p = 0.11). BCVA was significantly related to total NEI-VFQ-25 score and to 3 of the 12 NEI-VFQ-25 subscale scores (near activities: p = 0.01, role difficulties: p < 0.01 and driving: p < 0.01). Being a man was associated with higher NEI-VFQ-25 driving score (p < 0.01).

**Table 3:** Analysis of Glaucoma Symptom Scale (GSS) and 25-item National Eye Institute Visual Function Questionnaire (NEI-VFQ-25) scores at baseline by covariates (multivariable model).

N = 151	Age (for 10-year increase)		Sex (ref. female)		Baseline stage^a^			Baseline BCVA (≤0.15 vs >0.15 LogMar)	
	Slope/OR [95% CI]	P-value F-test	Slope/OR [95% CI]	P-value F-test	Early vs Severe	Moderate vs Severe	P-value F-test	Slope/OR [95% CI]	P-value F-test
					Slope/OR [95% CI]	Slope/OR [95% CI]			
**Glaucoma Symptom Scale (GSS)** ^**b**^									
Symp-6	1.01 [−1.30; 3.32]	0.39	4.01 [−2.33;10.36]	0.21	8.99 [−0.16;18.14]	10.43 [0.84;20.02]	0.09	6.31 [−2.80;15.41]	0.17
Func-4	0.11 [−2.12; 2.34]	0.93	***7.00 [0.86;13.13]***	***0.03***	***20.77 [11.93;29.62]***	***20.51 [11.24;29.78]***	**<** ***0.01***	8.02 [−0.78;16.82]	0.07
Total score	0.60 [−1.32; 2.53]	0.54	5.11 [−0.19;10.40]	0.06	***14.05 [6.41;21.68]***	***14.94 [6.93;22.94]***	**<** ***0.01***	7.43 [−0.17;15.03]	0.06
**National Eye Institute Visual Function Questionnaire (NEI-VFQ-25)** ^**b**^									
General health	−1.44 [−3.44; 0.56]	0.16	1.49 [−4.02; 7.00]	0.59	9.25 [1.30;17.19]	5.09 [−3.24;13.42]	0.06	1.11 [−6.79; 9.02]	0.78
General vision	−1.42 [−2.99; 0.15]	0.08	−2.26 [−6.57; 2.05]	0.30	***18.08 [11.86;24.29]***	***13.94 [7.42;20.45]***	**<** ***0.01***	3.09 [−3.10; 9.27]	0.33
Ocular pain	1.15 [−1.13; 3.43]	0.32	−0.04 [−6.30; 6.23]	0.99	7.08 [−1.95;16.11]	10.13 [0.67;19.60]	0.11	6.89 [−2.09;15.88]	0.13
Near activities	0.30 [−1.50; 2.10]	0.74	1.94 [−3.00; 6.89]	0.44	***16.72 [9.59;23.86]***	***16.18 [8.70;23.66]***	**<** ***0.01***	***10.07 [2.97;17.17]***	***0.01***
*Distance activities* ^*c*^	0.85 [0.65; 1.11]	0.22	1.49 [0.73; 3.06]	0.27	***19.37 [4.04;92.86]***	***19.18 [3.81;96.41]***	**<** ***0.01***	1.85 [0.64; 5.31]	0.25
*Social functionig* ^*c*^	1.09 [0.75; 1.59]	0.65	2.87 [0.93; 8.92]	0.07	***15.11 [4.04;56.51]***	***10.94 [2.87;41.69]***	**<** ***0.01***	2.02 [0.54; 7.63]	0.30
Mental health	0.82 [−1.03; 2.66]	0.39	1.26 [−3.82; 6.33]	0.63	***23.21 [15.89;30.53]***	***18.77 [11.10;26.44]***	**<** ***0.01***	3.79 [−3.50;11.07]	0.31
*Role difficulties* ^*c*^	1.14 [0.87; 1.50]	0.34	1.42 [0.66; 3.05]	0.37	***4.13 [1.49;11.44]***	***4.58 [1.52;13.79]***	***0.01***	***4.65 [1.65;13.08]***	**<** ***0.01***
*Dependency* ^*c*^	0.85 [0.59; 1.22]	0.38	1.02 [0.40; 2.57]	0.97	***19.90 [5.61;70.57]***	***7.00 [2.23;22.02]***	**<** ***0.01***	2.46 [0.81; 7.51]	0.11
Driving^d^	−1.09 [−2.96; 0.78]	0.25	***10.63 [5.54;15.72]***	**<** ***0.01***	***11.52 [4.39;18.65]***	***8.95 [1.29;16.61]***	***0.01***	***14.09 [6.43;21.76]***	**<** ***0.01***
*Color vision* ^*c*^	0.97 [0.56; 1.69]	0.92	4.03 [0.82;19.82]	0.09	***36.68 [3.77;357.3]***	***13.50 [2.22;82.17]***	**<** ***0.01***	1.52 [0.27; 8.60]	0.63
*Peripheral vision* ^*c*^	0.81 [0.59; 1.12]	0.21	1.83 [0.77; 4.35]	0.17	***9.85 [3.25;29.89]***	***8.84 [2.75;28.40]***	**<** ***0.01***	1.61 [0.54; 4.84]	0.39
Total score	0.01 [−1.08; 1.10]	0.98	1.77 [−1.24; 4.77]	0.25	***14.88 [10.55;19.21]***	***13.63 [9.09;18.17]***	**<** ***0.01***	***5.93 [1.62;10.24]***	***0.01***

Legend: N: Number of participants; BCVA: Best Correct Visual Acuity; OR: Odds Ratio; CI: Confidence Interval; ^a^Stage = Early (0/borderline/1); Moderate (2/3); Severe (4/5) ^b^Linear or logistic regression models (modeled the probability of score = “100”); ^c^Dichotomous score (‘ = 100’, ‘<100’). ^d^Data available for 111 patients; Bold and italic: Significant cvalues.

### Vision-related QoL and symptom score change over time

**Figure 1:**
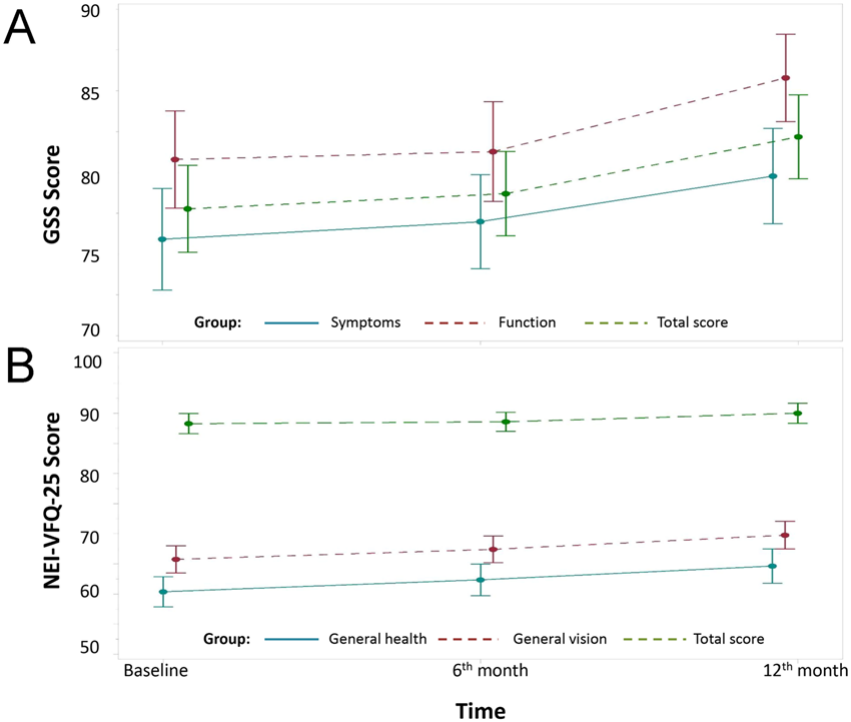
Trends over time of Glaucoma Symptom Scale (GSS) and 25-item National Eye Institute Visual Function Questionnaire (NEI-VFQ-25) scores. (**A**) Trends over time of GSS total score, Function-4 and Symptoms-6 subscale scores; (**B**) Trends over time of NEI-VFQ-25 total score, general health and general vision subscale scores.

GSS and NEI-VFQ-25 scores at baseline and at all follow-up visits are presented in Table 4. Mean baseline scores were higher than 75 in all the subscales, with the exception of NEI-VFQ-25 general health (60.4, SD 17.0), and NEI-VFQ-25 general vision (65.8, SD 15.4) scores. Color vision had the highest score (94.2, SD 8.0), followed by social functioning (97.7, SD 6.9) and dependency (95.5, SD 12.6). NEI-VFQ-25 and GSS total scores were 88.3 (SD 11.3) and 78.1 (SD 18.0), respectively. Over the 1-year follow-up, both the NEI-VFQ-25 and the GSS scores increased. The increase of all the GSS scores, the NEI-VFQ-25 total score and 7 of the 12 NEI-VFQ-25 subscale scores (general health, general vision, ocular pain, near activities, mental health, role difficulties and driving) was significant. Trends over time for the GSS and the NEI-VFQ-25 total scores and for the main subscale scores are presented in Fig. 1.

**Table 4:** Glaucoma Symptom Scale (GSS) and 25-item National Eye Institute Visual Function Questionnaire (NEI-VFQ-25) scores by visit.

Score	Baseline N = 178 Mean (SD)	6-month N = 178 Mean (SD)	12-month N = 178 Mean (SD)	Mixed models^a^	
				Slopes/OR (for 6-month increase) [CI]	P-value
**Glaucoma Symptoms Scale**					
Symp-6	76.2 (21.1)	77.0 (19.6)	79.8 (19.9)	1.81 [0.55; 3.08]	<0.01*
Func-4	81.2 (20.1)	81.3 (20.8)	85.8 (18.2)	2.32 [0.99; 3.64]	<0.01*
Total Score	78.1 (18.0)	78.7 (17.6)	82.2 (17.5)	2.06 [1.03; 3.10]	<0.01*
**National Eye Institute Visual Function Questionnaire (NEI-VFQ-25)**					
General health	60.4 (17.0)	62.3 (17.9)	64.6 (19.4)	2.13 [0.99; 3.28]	<0.01*
General vision	65.8 (15.4)	67.4 (15.0)	69.8 (15.7)	2.01 [0.89; 3.13]	<0.01*
Ocular pain	79.5 (19.9)	81.3 (17.6)	83.3 (18.5)	1.90 [0.57; 3.22]	<0.01*
Near activities	86.2 (17.4)	87.8 (15.8)	89.8 (14.7)	1.80 [0.69; 2.91]	<0.01*
Distance activities^b^–n (%) (100)	92 (51.7)	93 (52.3)	98 (55.1)	1.24 [0.80; 1.91]	0.33
Social functioning^b^–n (%) (100)	155 (87.1)	147 (82.6)	154 (86.5)	0.96 [0.63; 1.45]	0.83
Mental health	83.8 (18.2)	82.7 (18.8)	86.0 (17.4)	1.14 [0.15; 2.14]	0.03*
Role difficulties^b^–n (%) (100)	123 (69.1)	121 (68.0)	137 (77.0)	1.80 [1.06; 3.06]	0.03*
Dependency^b^ – n (%) (100)	144 (80.9)	142 (79.8)	152 (85.4)	1.58 [0.85; 2.94]	0.15
Driving^c^	87.8 (16.4)	88.5 (16.2)	90.6 (14.8)	0.99 [0.03; 1.94]	0.04*
Color vision^b^–n (%) (100)	168 (94.4)	166 (93.3)	163 (91.6)	0.93 [0.29; 2.94]	0.90
Peripheral vision^b^–n (%) (100)	140 (78.7)	130 (73.0)	139 (78.1)	1.32 [0.71; 2.48]	0.38
Total Score	88.3 (11.3)	88.6 (10.7)	90.0 (11.2)	0.87 [0.32; 1.41]	<0.01*

Legend: N: Number of participants; OR: Odds Ratio; CI: Confidence Interval; ^a^Longitudinal linear mixed model or logistic longitudinal mixed model (modeled the probability of score = “100”); ^b^Dichotomous score (‘ = 100’, ‘<100’); ^c^Data available for 145 patients. *Significant values.

**Figure 2:**
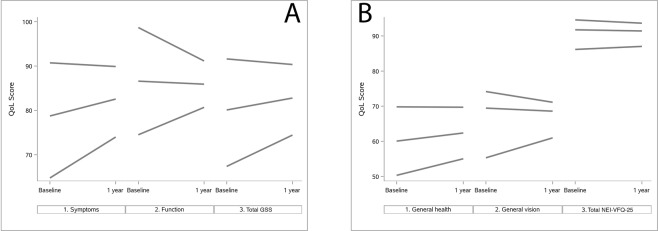
Estimated quality of life scores by time and covariates (multivariable analysis). (**A**) Estimated GSS Symp-6 (Symptoms), Func-4 (Function) and Total score variations over time, according to score at baseline; (**B**) Estimated NEI-VFQ-25 General Health, General Vision and Total score variations over time, according to score at baseline.

**Figure 3:**
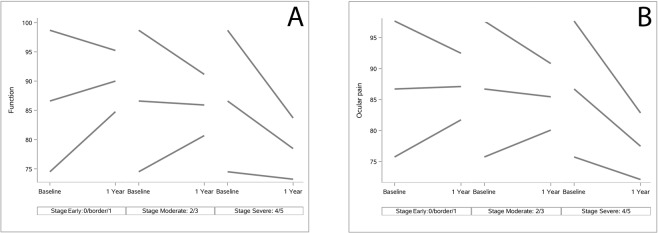
Estimated quality of life scores by time and covariates (multivariable analysis). (**A**) Estimated GSS Func-4 score variation over time, according to score at baseline and baseline stage of the disease; (**B**) Estimated NEI-VFQ-25 Ocular Pain score variation over time, according to score at baseline and baseline stage of the disease.

**Figure 4:**
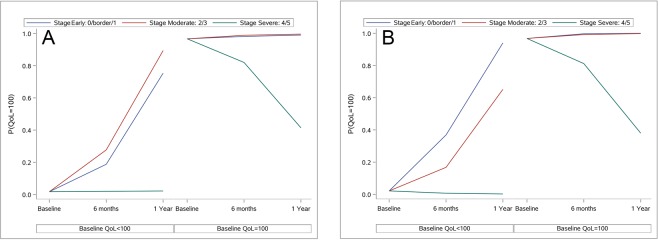
Estimated probability to achieve maximum qualitative QoL score (100) by time and covariates. (**A**) Probability over time to achieve NEI-VFQ-25 Vision-specific Role Difficulties maximum score (100), according to score at baseline and baseline stage of the disease; (**B**) Probability to achieve NEI-VFQ-25 Peripheral Vision maximum score (100), according to score at baseline and baseline stage of the disease.

Results of multivariable analyses on NEI-VFQ-25 and GSS scores over time are reported in Table 5. A significant negative interaction was observed between time and all the GSS scores, the NEI-VFQ-25 total score and 8 of the 12 NEI-VFQ-25 subscale scores at baseline (general health, general vision, ocular pain, near activities, social functioning, mental health, role difficulties and dependency). As a result, a lower baseline score was related to a higher increase of the score during the follow-up (Fig. 2). A negative interaction between time and baseline stage of the disease was found for GSS Func-4 score (p < 0.01) and NEI-VFQ-25 ocular pain score (p = 0.02) (Fig. 3). In thise case, lower glaucoma stages at baseline (i.e. early or moderate disease) were associated with higher increase of QoL score over time. With regard to NEI-VFQ-25 dichotomized scores, a significant interaction between stage of the disease and time was detected on vision-specific role difficulties (p = 0.01, Fig. 4A) and peripheral vision (p = 0.016, Fig. 4B, available online) scores. This means that patients without a maximum score (<100) and with an early to moderate disease at baseline, had a high probability to achieve the maximum score during the follow-up. Patients with a maximum QoL score (100) at baseline and a severe disease, more easily experienced a QoL score reduction over time.

**Table 5:** Analysis of Glaucoma Symptom Scale (GSS) and 25-item National Eye Institute Visual Function Questionnaire (NEI-VFQ-25) scores by time, covariates and their interactions (multivariable model).

N = 150	P-valueBaseline QoL score*time	P-value Sex*time	P-value Age*time	P-value ^a^Stage*time	P-value BCVA*time	P-value Treat*time
**Glaucoma Symptom Scale (GSS)**						
Symp-6	**<0.001**	0.673	0.594	0.113	0.950	0.619
Func-4	**<0.001**	0.256	0.759	**0.009**	0.333	0.940
GSS score	**<0.001**	0.561	0.855	0.108	0.984	0.921
**National Eye Institute Visual Function Questionnaire (NEI-VFQ-25)**						
General health	**0.001**	0.345	0.248	0.759	0.186	0.239
General vision	**<0.001**	0.057	0.192	0.908	0.147	0.528
Ocular pain	**<0.001**	0.176	0.321	**0.027**	0.321	0.768
Near activities	**<0.001**	0.434	0.295	0.495	0.329	0.526
Distance activities^b^	0.764	0.068	0.508	0.248	0.843	0.076
Social functioning^b^	**<0.001**	0.907	0.690	0.172	0.760	0.054
Mental health	**<0.001**	0.262	0.643	0.269	0.701	0.203
Role difficulties^b^	**0.027**	0.992	0.390	**0.010**	0.717	0.188
Dependency^b^	**0.024**	0.425	0.544	0.127	0.459	0.927
Driving^c^	0.069	0.565	0.518	0.706	0.391	0.906
Color vision^b^	0.851	0.380	0.114	0.194	0.580	0.704
Peripheral vision^b^	0.595	0.781	0.658	**0.016**	0.836	0.352
Total score	**<0.001**	0.188	0.344	0.548	0.834	0.541

Legend: N: Number of participants; BCVA: Best corrected visual acuity (≤0.15 LogMar, >0.15 LogMar); Treat: Number of drugs administered (>1 vs 1); ^a^Stage = Early (0/borderline/1), Moderate (2/3), Severe (4/5); ^b^Dichotomous scores (‘ = 100’, ‘<100’); ^c^Data available for 111 patients; Bold: Significant values.

Model: Score = Baseline score, time, Baseline score*time, time*gender, time*age, time*baseline stage, time*BCVA, time*Treat.

## Discussion

Several cross-sectional studies have found a significant association between VF and BCVA deterioration and vision-related QoL in glaucoma patients”. However, only a few studies investigated QoL with a prospective design^23,24^. The present study aimed to evaluate vision-related QoL and glaucoma-related symptom perception both at baseline and prospectively, in a group of newly-diagnosed glaucoma patients followed-up for one year. As a result, GSS and NEI-VFQ-25 scores were generally high at baseline (i.e. >75), indicating that this population of newly-diagnosed glaucoma patients perceived a high level of QoL. Stage of the disease and BCVA were the most important determinants of QoL at baseline. As GStag2 resides entirely on VFD, these findings are not surprising and are in agreement with previous cross-sectional studies, where a significant correlation between both VF and BCVA with QoL was found^9,10,23^. In a recent study analyzing QoL of patients enrolled in the Early Manifest Glaucoma Trial and followed-up for 20 years, visual acuity and VF index were the only significant variables influencing QoL, accounting for nearly 40% of NEI-VFQ-25 scores^9^. On the other hand, both visual acuity and VF scores were weakly but significantly associated with QoL measures in newly-diagnosed glaucoma patients enrolled in the CIGTS^10^. Beside these data, it is unknown if QoL in glaucoma is more affected by VFD or BCVA deterioration. In the present study, stage of the disease was associated with 2 of the 3 GSS scores at baseline, and with all the NEI-VFQ-25 scores, with the exception of general health and ocular pain. BCVA was associated with only 3 of the 12 NEI-VFQ-25 scores and with total NEI-VFQ-25 score. These results probably suggest a prominent role of VFD as a determinant of QoL in newly-diagnosed glaucoma patients, albeit further researches are needed to confirm these findings.

Men showed a higher perception of their visual function at baseline (GSS Func-4 score), and were more confident at driving than women (NEI-VFQ-25 driving score). Sex-related psychological profiles are likely involved in these results. Previous studies have found women to be more prone to reporting symptoms than men^45,46^ and to engage more easily in illness behaviors^46^. Nevertheless, anxiety and depression, two common responses to the diagnosis of a chronic illness^47^ are more common in women than men^48–50^. Our results about driving are in agreement with the conclusions of previous studies, where women with glaucoma were more likely to cease driving than men^18,51^.

In our study NEI-VFQ-25 total score and most of the NEI-VFQ-25 subscale scores significantly increased from baseline to the 1-year follow-up visit, which suggests a general pattern of improvement in vision-related QoL. Similarly, the GSS total score as well as symptom and function subscale scores improved, which suggests a blunting of glaucoma-related symptom perception over time. Reason of this change is unknown, however adaptation to glaucoma diagnosis may be involved. Adaptation to a chronic illness is a well-known phenomenon in general medicine, and has been extensively investigated^52–55^. Several reports from different disciplines have highlighted the role of adaptation to the diagnosis and its impact on QoL of various chronic diseases and disabilities^56–58^. Adaptation theories have been also translated into clinically meaningful rehabilitation programs^57^. Little is known about the effects of adaptation to the diagnosis of glaucoma, and a few studies have investigated this phenomenon. Interestingly, in many participants of the CIGTS, the diagnosis of glaucoma engendered an immediate response of fear of blindness^59^. However, the percentage of respondents who reported moderate or significant apprehension dropped from 34% at baseline to less than 15% after 12 months. In the same study, a slight but significant decline in symptom scale scores was recorded during the follow-up, indicating an improvement in the perception of glaucoma-related symptoms^23^. Psychological adaptation to the diagnosis of glaucoma was offered as an explanation of these results, while less convincing was the hypothesis of a diminution or cessation of the particular problem.

Two variables greatly influenced QoL score change over time in our study: QoL score at baseline and stage of the disease. While the interaction of time with baseline QoL scores was significant over most GSS and NEI-VFQ-25 scores, the interaction of baseline stage with time was significant only for GSS Function-4 score and NEI-VFQ-25 ocular pain, role difficulties and peripheral vision scores. According with this analysis, higher QoL score increase over time was modeled in patients with lower QoL score at baseline and less advanced disease. Both psychological and clinical factors are likely to be involved in these results. Psychological impact of the diagnosis of a chronic illness is quite different according to personality traits and life expectation^60,61^. The process of adaptation may mitigate the initial response over time, and a greater increase in QoL scores may be expected in patients that initially negatively reacted to the diagnosis. While psychological factors may be alleviated over time, functional factors persist, and their effect on QoL is likely to be permanent. As a consequence, patients with severe disease at baseline had a trend towards a stabilization or a reduction of their QoL scores over time, irrespectively of score values at baseline.

All the patients included in this study were administered topical hypotensive medications after the baseline visit. Moreover, 27 patients underwent laser treatment and 8 patients underwent surgery during the follow-up. Glaucoma medications may affect QoL, due to the local side effects (burning, stinging, blurred vision, etc.), and the bothersomeness of time-fixed administrations, potentially interfering with daily routine^62,63^. In our study a trend towards an increase in the number of topical agents per patient was found over time. This is not surprising, as it’s common practice to initiate glaucoma therapy with a single agent and add further medications if target IOP is not achieved. Despite the raise in medication number, QoL scores increased over the follow-up, including the GSS Symp-6 score, that specifically evaluates local symptoms (burning/smarting/stinging, tearing, dryness, itching, soreness/tiredness, feeling of something in the eye). Moreover, when patients were dichotomized according to the number of topical treatments received (one vs. more than one), no significant interaction with time was found. These results are somehow unexpected. However, it is possible that the mechanism of adaptation may be valid not only for the psychological burden of a new diagnosis, but also for the symptoms of the disease and its treatment^23^.

Our study has a number of limitations. First, no rigorous methodology for VF progression evaluation was adopted, as the follow-up was considered too short for a such analysis. However, taking into account the mild VFD at baseline (mean MD: −4.5 ± 5.3), it is unlikely that VF changes had an effect on QoL, with the obvious exception of patients with very fast progression and very high IOP. Second, data from both eyes were included in the analysis, when available. It is debated if vision-related QoL in glaucoma patients is more dependent on the patients’ better eye, worse eye or a combination thereof^9,17,27,64^. The decision to include data from both eyes of participants with bilateral disease was made in an attempt to obtain a model that reflects the real-life perception of patients as closely as possible. Third, patients were treated at physician’s discretion during the follow-up, in order to delay the progression of the disease. A precise evaluation of QoL and symptoms in surgically-treated patients was not feasible, due to the low number of patients that underwent surgery during the follow-up. On the other hand, all the patients receiving laser treatment were simultaneously treated with topical medical therapy, so it is reasonable to suppose that QoL and symptoms in this group were similar to the rest of the studied population.

In conclusion, this study has demonstrated an increase in vision-related QoL scores and a reduction in glaucoma-related symptom perception over one-year follow-up in a population of newly-diagnosed glaucoma patients. Although the precise explanation for these findings is uncertain, psychological processes and adaptation to the diagnosis of a chronic illness might have played a role. Future research is warranted in order to evaluate possible changes of vision-related QoL in a larger sample of glaucoma patients with a more extended follow-up.

## Data Availability

The datasets generated during and/or analyzed during the current study are available from the corresponding author on reasonable request.
