# Author Correction: Vision-related quality of life and symptom perception change over time in newly-diagnosed primary open angle glaucoma patients

**DOI:** 10.1038/s41598-020-60489-2

**Published:** 2020-03-06

**Authors:** Ivano Riva, Lorenzo Legramandi, Eliana Rulli, Anastasios G. Konstas, Andreas Katsanos, Francesco Oddone, Robert N. Weinreb, Luciano Quaranta, The Italian Study Group on QoL in Glaucoma*, L. Varano, T. Carchedi, S. Talarico, P. Frezzotti, F. Parravano, I. Motolese, S. A. Bagaglia, G. C. M. Rossi, S. Lateri, L. Bossolesi, L. Carmassi, T. Rolle, R. Piccini, R. Ratiglia, A. Rossi, S. Gandolfi, V. Tagliavini, N. Ungaro, M. Fossarello, A. Cuccu, I. Zucca, M. Uva, E. Bonacci, G. Cardarella, D. Tognetto, O. Vattovani, P. Vallon, F. Iannacone, L. Fontana, S. Marchi, G. L. Manni, D. Jannetta, G. Roberti, L. Rossetti, E. Maggiolo, O. Oneta, C. Sborgia, F. Cantatore, L. Mastropasqua, L. Agnifili, E. Campos, C. Gizzi, G. Giannaccare, V. Pucci, M. Cassamali, C. Costagliola, C. Traverso, R. Scotto, M. Musolino, L. Landi, A. Bagnis

**Affiliations:** 1 IRCCS - Fondazione Bietti, Rome, Italy; 2 IRCCS - Istituto di Ricerche Farmacologiche Mario Negri, Milan, Italy; 3 1st University Department of Ophthalmology, Aristotle University of Thessaloniki, Thessaloniki, Greece; 4 Department of Ophthalmology, University of Ioannina, Ioannina, Greece; 5 Hamilton Glaucoma Center, Shiley Eye Institute, and the Department of Ophthalmology, University of California San Diego, San Diego, USA; 6 Department of Surgical & Clinical, Diagnostic and Pediatric Sciences, Section of Ophthalmology, University of Pavia - IRCCS Fondazione Policlinico, San Matteo, Pavia Italy; 7 Università degli Studi “Magna Graecia”, Catanzaro, Italy; 8 A.O.U. Senese Ospedale Santa Maria delle Scotte, Università di Siena, Siena, Italy; 9 Fondazione IRCCS Istituto di Ricovero e Cura a Carattere Scientifico Policlinico San Matteo, Pavia, Italy; 10 IRCCS Istituto di Ricovero e Cura a Carattere Scientifico Istituto Auxologico Italiano, Milano, Italy; 11 Università degli Studi di Torino, Torino, Italy; 12 Fondazione IRCCS Istituto di Ricovero e Cura a Carattere Scientifico Ca’ Granda Ospedale Maggiore Policlinico, Milano, Italy; 13 A.O.U. di Parma, Parma, Italy; 14 A.O.U. Cagliari - Ospedale Civile San Giovanni di Dio, Cagliari, Italy; 15 A.O.U. “Policlinico Vittorio Emanuele” P.O. Gaspare Rodolico, Catania, Italy; 16 A.O.U. “Ospedale Maggiore”, Trieste, Italy; 17 A.O. Arcispedale Santa Maria Nuova-IRCCS, Reggio Emilia, Italy; 18 Università Tor Vergata, Fondazione Policlinico Tor Vergata, Roma, Italy; 19 A.O. S. Paolo, Milano, Italy; 20 A.O.U. Policlinico, Bari, Italy; 21 Ospedale Clinicizzato SS. Annunziata, Chieti, Italy; 22 A.O.U. Policlinico S. Orsola Malpighi, Bologna, Italy; 23 A.O. di Desenzano del Garda, Desenzano del Garda, Brescia, Italy; 24 Dipartimento SPES Università del Molise, Campobasso, Italy; 25 IRCCS AOU San Martino - IST, Genova, Italy

Correction to: *Scientific Reports* 10.1038/s41598-019-43203-9, published online 01 May 2019

Paolo Frezzotti was omitted from The Italian Study Group on QoL in Glaucoma in the original version of this Article.

In addition, the original version of this Article contained errors in the spelling of the authors V. Tagliavini and V. Pucci which were incorrectly given as Tagliavini and Pucci respectively

The Italian Study Group on QoL in Glaucoma now reads as:

“L. Varano, T. Carchedi, S. Talarico, P. Frezzotti, F. Parravano, I. Motolese, S. A. Bagaglia, G. C. M. Rossi, S. Lateri, L. Bossolesi, L. Carmassi, T. Rolle, R. Piccini, R. Ratiglia, A. Rossi, S. Gandolfi, V. Tagliavini, N. Ungaro, M. Fossarello, A. Cuccu, I. Zucca, M. Uva, E. Bonacci, G. Cardarella, D. Tognetto, O. Vattovani, P. Vallon, F. Iannacone, L. Fontana, S. Marchi, G. L. Manni, D. Jannetta, G. Roberti, L. Rossetti, E. Maggiolo, O. Oneta, C. Sborgia, F. Cantatore, L. Mastropasqua, L. Agnifili, E. Campos, C. Gizzi, G. Giannaccare, V. Pucci, M. Cassamali, C. Costagliola, C. Traverso, R. Scotto, M. Musolino, L. Landi & A. Bagnis”

This has been corrected in the PDF and HTML versions of the Article.

